# Pathogenic Mutations in the Tumor Microenvironment Drive Tumor Progression in Diffuse Large B-Cell Lymphoma Through Tumor–Stroma Cross-Talk

**DOI:** 10.3390/cancers18111697

**Published:** 2026-05-22

**Authors:** Vaishali Aggarwal, Radhika Srinivasan, Amanjit Bal, Pankaj Malhotra, Subhash Varma, Ashim Das

**Affiliations:** 1 Molecular Biology Laboratory, Department of Histopathology, Post Graduate Institute of Medical Education and Research (PGIMER), Chandigarh 160012, India; vaishali.pgi@gmail.com (V.A.); docaman5@hotmail.com (A.B.); 2 Department of Immunology, School of Medicine, University of Pittsburgh, Pittsburgh, PA 15213, USA; 3 Tumor Microenvironment Center, UPMC Hillman Cancer Center, Pittsburgh, PA 15213, USA; 4 Department of Cytology and Gynaecological Pathology, Post Graduate Institute of Medical Education and Research (PGIMER), Chandigarh 160012, India; drsradhika@gmail.com; 5 Department of Clinical Hematology and Medical Oncology, Post Graduate Institute of Medical Education and Research (PGIMER), Chandigarh 160012, India; malhotrapankaj@hotmail.com (P.M.); suvarma@gmail.com (S.V.); 6 Internal Medicine, Fortis Hospital, Mohali 160062, India; 7 Department of Hepatopathology, Kalinga Institute of Medical Sciences (KIMS), Bhubaneswar 751024, India

**Keywords:** tumor microenvironment, gene mutations in stromal/extracellular matrix, tumor–stroma crosstalk, cytokine, DLBCL

## Abstract

**Background**: Diffuse Large B-cell Lymphoma (DLBCL) is a biologically heterogeneous subtype of non-Hodgkin’s lymphoma (NHL), accounting for 30–40% of cases worldwide. Despite the incorporation of rituximab into standard chemo-immunotherapy regimen, approximately one-third of patients present with relapsed or refractory disease, implicating the need for improved prognostic markers and therapeutic targets. Gene expression profiling successfully classified DLBCL into Germinal Center B-cell-like (GCB) and non-GCB subtypes, which differ in genetic alterations, response to therapy, and clinical outcome. While intrinsic tumor biology has been extensively studied, the contribution of the tumor microenvironment (TME) to disease progression and therapeutic resistance still remains incompletely understood. **Methods**: In this study, we investigated the mutational landscape of stromal-related genes in DLBCL and evaluated their impact on gene expression, downstream signaling pathways, and tumor progression. **Results**: A total of 176 DLBCL patients were screened, of which 113 were enrolled based on availability of complete clinical data. The cohort demonstrated male predominance (male:female ratio: 2.1:1), advanced disease stage in 72.6% of patients, and elevated serum lactate dehydrogenase levels in 57.5%. Based on immunohistochemistry, 43.4% cases were classified as GCB-DLBCL and 56.6% as non-GCB DLBCL. Although the International Prognostic Index (IPI) retained prognostic significance for event-free survival (EFS) and overall survival (OS), considerable heterogeneity was observed within similar risk groups. Whole-exome sequencing (WES) uncovered recurrent somatic mutations in key oncogenic and epigenetic regulators, including TNFAIP3, NFIB, NOTCH1, TSC2, EZH2, EP300, KMT2D, and B2M, with subtype-specific distribution. Pathway enrichment analysis implicated role of Notch, Wnt, mTOR, JAK-STAT, TGF-β, and antigen-presentation pathways. Comprehensive WES analysis identified multiple novel mutations in genes associated with the stromal/extracellular matrix with distinct patterns in GCB and non-GCB DLBCL, accompanied by concordant alterations in gene expression profiles, suggesting functional relevance within the TME. Functional validation through primary cell culture demonstrated significantly elevated Th2 (IL-4, IL-6, IL-10) and Th17 (IL-17) cytokines in co-cultures containing both neoplastic cells and stromal components, underscoring the role of TME in DLBCL progression. **Conclusions**: Taken together, this study provides novel insights into stromal mutational signatures and cytokine-mediated tumor–stroma interactions, offering potential prognostic biomarkers and therapeutic targets for the improved management of DLBCL.

## Simple Summary

Biological heterogeneity in Diffuse Large B-cell Lymphoma (DLBCL) accounts for variable therapeutic response to established rituximab plus standard chemo-immunotherapy regimens, which are attributed to one-third of patients presenting with relapsed and refractory DLBCL. This warrants further investigation for improved prognostic and therapeutic targets in clinical settings. Though tumor intrinsic biology is extensively studied in DLBCL, the contribution of the tumor microenvironment (TME) to disease progression and therapeutic resistance still remains incompletely understood. This study highlights the importance of stromal gene signatures as prognostic markers in DLBCL. We propose differential stromal gene markers for Germinal Center B-cell-like (GCB) and non-GCB DLBCL with novel mutations in genes associated with the stromal/extracellular matrix and prognostic significance, which would need further exploration for translation into a clinical setting. Taken together, this study provides novel insights into stromal mutational signatures and cytokine-mediated tumor–stroma interactions, offering potential prognostic biomarkers and therapeutic targets for the improved management of DLBCL.

## 1. Introduction

Diffuse Large B-cell Lymphoma (DLBCL) is a highly heterogeneous and aggressive form of non-Hodgkin’s lymphoma (NHL) [1,2,3,4,5]. Despite the introduction of rituximab in the therapeutic armamentarium, recent therapeutic advancements to improve overall survival of DLBCL cases has been challenging with roughly one-third of cases presenting with relapsed/refractory DLBCL [6,7,8,9]. Two major DLBCL subtypes were discerned using gene expression profiling based upon presumptive cell of origin (COO) classification including the Germinal Center B-cell (GCB) and the non-GCB phenotype, each associated with differential clinical outcomes and specific genetic alterations [10,11]. In contrast to GCB-DLBCL, non-GCB DLBCL is more aggressive and has a poor response to chemotherapy and overall survival (OS), mainly attributed to the aberrant activation of the *NF-κB* pathway in the non-GCB phenotype [9,12]. With a high relapse rate and poor prognosis reported in DLBCL patients, the focus for delineating new therapeutic targets has shifted from tumor-specific pathways to TME in somatic cancers [13,14]. The importance of this theory was reciprocated in a landmark study by Lenz et al. [15] using a gene expression profiling platform in which the authors identified two differential stromal coding prognostic signatures, such as stromal-1/stromal-2 signatures in DLBCL. Stromal-1 and stromal-2 gene signatures elucidated biologic variables that dictated a response to chemotherapy in DLBCL patients and predicted survival in a statistically independent manner [15,16]. Of these, the stromal-1 signature, rich in components of extracellular matrix and histiocytic infiltration, is associated with better survival and good prognosis, whereas the stromal-2 signature, categorized by angiogenesis markers, is associated with reduced survival and poor prognosis in DLBCL patients. Following the initial excerpt published in 2008 by Lenz et al. [15], recent studies have also supported a gene expression profiling-based predictor model for DLBCL cases [17,18,19]. Besides these major studies, there are very few studies documenting the molecular basis of TME in DLBCL progression, and hence, it is still the area of great potential leading to novel therapeutic targets of stromal cells for better management and treatment of DLBCL patients [12,20,21,22].

In view of the limited scientific literature, this exploratory study investigates the mutation profile of stromal-related genes in DLBCL cases and its effect on expression profiling and downstream targets. Furthermore, the potential role of TME on tumor progression was also evaluated through primary cell culture of DLBCL cases, which was further corroborated with the cytokine expression in vivo.

## 2. Materials and Methods

This study was approved by the Institute Ethics Committee (IEC) of PGIMER (protocol code IEC-08/2018-959 dated 26 December 2018) and conducted in accordance with the Declaration of Helsinki. Informed consent was obtained from all the patients enrolled in this study. A total of 176 DLBCL patients were screened, of which 113 were enrolled based on availability of complete clinical data. The cohort demonstrated male predominance (male:female ratio: 2.1:1), advanced-stage disease in 72.6% of patients, and elevated serum lactate dehydrogenase levels in 57.5%. Based on immunohistochemistry, 43.4% cases were classified as GCB-DLBCL and 56.6% as non-GCB DLBCL (Supplementary Figure S1). A pilot whole exome sequencing (WES) analysis was carried out in four cases of DLBCL as per the WHO (World Health Organisation) diagnostic criteria for primary nodal DLBCL, along with two cases of reactive lymph nodes as controls followed by Sanger sequencing in a validation cohort of 25 DLBCL cases. All the potential mutations in genes associated with the stromal/extracellular matrix identified from WES were validated using Sanger sequencing. Real-time expression profiling was carried out in 24 cases to study the expression profile of mutated stromal genes. A total of five cases of DLBCL were subjected to primary cell culture to evaluate the effect of TME on tumor cells by evaluating the Th1/Th2/Th17 cytokine profile.

### 2.1. Whole Exome Sequencing (WES)

A pilot WES was carried out on a total of six lymph node biopsy patient samples (fresh, frozen), including GCB, and non-GCB DLBCL (two cases each) classified immunohistochemically based on Han’s algorithm. Reactive lymphoid hyperplasia (two cases) as controls were sequenced using next-generation sequencing platform Ion Proton™ (Life Technologies, ThermoFisher Scientific, Waltham, MA, USA) to perform WES. DNA was isolated using a Pure Link^®^Pro 96 Genomic DNA Purification kit (K182104A, Invitrogen, ThermoFisher Scientific, USA), subsequently processed for qualitative checks on a 2100 Agilent Bioanalyzer (G2940CA, Agilent Technologies, Santa Clara, CA, USA), followed by library preparation, genomic exon enrichment, and template amplification using an Ion Ampliseq^TM^ Exome kit (Life Technologies, ThermoFischer Scientific, USA). Sequencing was performed on Ion Proton^TM^ (ThermoFischer Scientific, USA). Filtered reads were mapped to the hg19: reference human genome using Torrent Suite™ Software v5.2.2, after which Torrent Variant Caller (TVC) v5.2.2 software was used to analyze for variant calling, and data were further analyzed using Ion Reporter^TM^ Software 5.20. Reference genome was mapped to tumor sample for the pipeline analysis of mutations including filtering out common SNPs (UCSC genome browser), and the inclusion of variants in COSMIC and dbSNP, followed by functional scores for Polyphen (0.15–1.0) and SIFT < 0.05. The variants were then visualized using the Integrative Genomics Viewer platform (IGV, version 2.19.7). The pathogenic variants identified using pipeline analysis were evaluated further for associated biological pathways bioinformatically using the PANTHER classification system (http://www.pantherdb.org, accessed on 15 May 2026), DAVID Bioinformatics Resources (https://davidbioinformatics.nih.gov/home.jsp, accessed on 15 May 2026), PCViz (http://www.pathwaycommons.org/pcviz, accessed on 15 May 2026), and STRING (https://string-db.org, accessed on 15 May 2026).

### 2.2. Sanger Sequencing

Sanger sequencing was carried out for the validation of identified mutations from WES in an independent validation cohort of DLBCL patients (n = 25). Primer3plus was utilized for primer designs for chromosomal coordinates harboring potential mutations as regions of interest and revalidated using Gene Runner software v6.0 and detailed in Supplementary Table S1. DNA sequencing (Sanger sequencing) was performed for both strands on the amplified PCR products using BigDye terminator kits (ABI PRISM 3130 analyzer, Applied Biosystems, Waltham, MA, USA). FinchTv was used to visualize sequencing data (PerkinElmer, Shelton, CT, USA) followed by analysis through CodonCode Aligner (v 6.0.2, Centerville, MA, USA).

### 2.3. Gene Expression by PCR Array for Stromal Genes

The PCR array was customized for stromal gene expression using the RT^2^ profiler PCR array detailed in Supplementary Figure S2 (CAPH-13454C, Qiagen, Germantown, MD, USA). A total of 44 genes were included in the plate set-up design for quantitative expression analysis, including two house-keeping genes (18D and ACTB), 42 test genes, a non-template control (RTC), and genomic DNA contamination control (HGDC). Panel included mutated stromal genes shortlisted from the exome data including genes coding for mucin, laminin, collagen, receptors, and extracellular matrix structural proteins, along with genes coding for matrix metallo-proteinases, macrophages, endothelial cells, and cytokines. The quantitative experiments were performed on 24 DLBCL cases (12 cases each of GCB and non-CGB DLBCL) in triplicates with a cut-off of ±2 compared to controls (reactive lymphoid hyperplasia cases).

The RNeasy FFPE kit was used to isolate RNA from FFPE samples (73504, Qiagen, USA), quantitated using NanoDrop (NanoDrop 2000, ThermoScientific, Waltham, MA, USA), followed by quality checks using the Agilent 2100 Bioanalyzer (Agilent^®^ Technologies, USA) for the A260/A280 ratio for RNA purity. The RT^2^first strand kit was used for cDNA synthesis (330401, Qiagen, USA), followed by a real-time polymerase chain reaction (RT-PCR) utilizing primers as illustrated in Supplementary Table S2, and executed on the Applied Biosystems StepOnePlus^TM^ platform (ThermoFisher Scientific, USA). A total of 50 ng of total RNA was used for the reverse transcription of each sample (Supplementary Table S3). RT-PCR cycling was performed with initial denaturation for 10 min at 95 °C, followed by 40 amplification cycles for 15 s at 95 °C, and a final extension for 1 min at 60 °C. RTC was run with each sample with a substitution of PCR-grade water for DNA, and each sample was run in triplicates. StepOnePlus^TM^ real-time cycler software v2.2 was used to estimate the threshold (C_T_) value (Applied Biosystems, ThermoFisher Scientific, USA). The “Delta-delta” C_T_ method was used to analyze Real Time PCR data to compare the relative expression of stromal genes in tumor versus normal samples.

### 2.4. OncoPrint Analysis of Gene Mutations Associated with the Stromal/Extracellular Matrix in the Publicly Available Whole Exome Sequencing DLBCL Dataset

We further evaluated the publicly available WES dataset of tumor/matching normal sample pairs for DLBCL patients (n = 135) from the dbGAP accession numbers phs000450 and GSE98588 [11] using cBioPortal [23,24]. cBioPortal is a publicly available web resource developed for exploring, visualizing, and analyzing multidimensional cancer genomics data. The cBioPortal platform provides an interactive user interface for interactively exploring genetic alterations across samples, genes, and pathways, and correlates to clinical outcomes [23,24]. The OncoPrint for mutated stromal genes also showed the genetic alteration type, oncotree code (GCB, non-GCB, or DLBCL NOS), IPI score, mutation spectrum, R-CHOP status, overall survival, progression free survival, and frequency of mutated stromal genes in DLBCL cases.

### 2.5. Study of Secreted Cytokine Profile in DLBCL Primary Cell Culture

Primary cell culture was carried out in five cases of DLBCL. A short culture time over a span of one week was consistently maintained with lymphoma cells in suspension. Explant culture was carried out on a part of fresh lymph node biopsy and processed in Gibco^®^ RPMI 1640 medium (Gibco, ThermoFisher Scientific, USA) with 20% heat-inactivated fetal bovine serum (FBS) (SigmaAldrich, St. Louis, MA, USA) and 1% Penicillin–Streptomycin (SigmaAldrich, USA), and incubation at 37 °C for 48 h in 5% CO_2_ (New Brunswick^TM^ Galaxy^®^170R, Eppendorf, Hamburg, Germany). Following 48 h culture, FACS sorting of cells was performed using CD19 PE-conjugated antibody (Bector Dickinson (BD) Biosciences, Franklin Lakes, NJ, USA). BD FACSAria^TM^ III was used to sort CD19^+^ tumor cells and CD19^−^ non-neoplastic cells (BD Biosciences, Franklin Lakes, NJ, USA). The cell density, cell viability, and culture duration as mentioned were maintained consistent across all five cases subjected to flow sorting. The sorted CD19^+^ and CD19^−^ cells were checked for purity and viability followed by re-culture and incubation for 24 h in 6-well plate, after which 1-well/6-well plates each of CD19^+^ and CD19^−^ cells were centrifuged for 5 min at 1500 rpm, leaving the remaining wells/6-well plate untouched. Post-centrifugation, the supernatant of CD19^+^ cells was decanted, and supernatant from CD19^−^ cells was transferred to CD19^+^ cells. The pellet was resuspended and re-cultured, marked as CD19^+/−^, and incubated for 48 h with similar culture media composition as described above. Post-incubation, supernatant from CD19^+^, CD19^−^, and CD19^+/−^ cells was collected and stored at −80 °C for analysis of Th1/Th2/Th17 cytokine profiles using the Human Th1/Th2/Th17 Cytokine Kit, BD^TM^Cytometric Bead Array (CBA) for quantitative cytokine analysis (Becton Dickinson, Biosciences, USA).

### 2.6. Cancer Genome Atlas (TCGA) Survival Analysis

TCGA survival analysis for identified stromal gene signatures was carried out using the UALCAN ‘Lymphoid neoplasm Diffuse Large B-Cell Lymphoma’ TCGA dataset [25]. UALCAN is a comprehensive, interactive web resource for analyzing cancer OMICS data (TCGA, MET500, CPTAC) for the identification of biomarkers. The ‘lymphoid neoplasm Diffuse Large B-Cell Lymphoma’ dataset contains survival data of DLBCL patients (n = 48).

### 2.7. Statistical Analysis

GraphPad Prism 5.0 and SPSS (version 22, SPSS, Chicago) were used for statistical analysis. Mann–Whitney (non-parametric test) and Pearson’s chi-square test were used to evaluate relative expression and cytokine profile. Two-tailed tests were performed and statistically significance was observed at * *p* < 0.05, ** *p* < 0.01, *** *p* < 0.001, and **** *p* < 0.001.

## 3. Results

### 3.1. Mutations in Genes Associated with the Stromal/Extracellular Matrix

**Figure 1:**
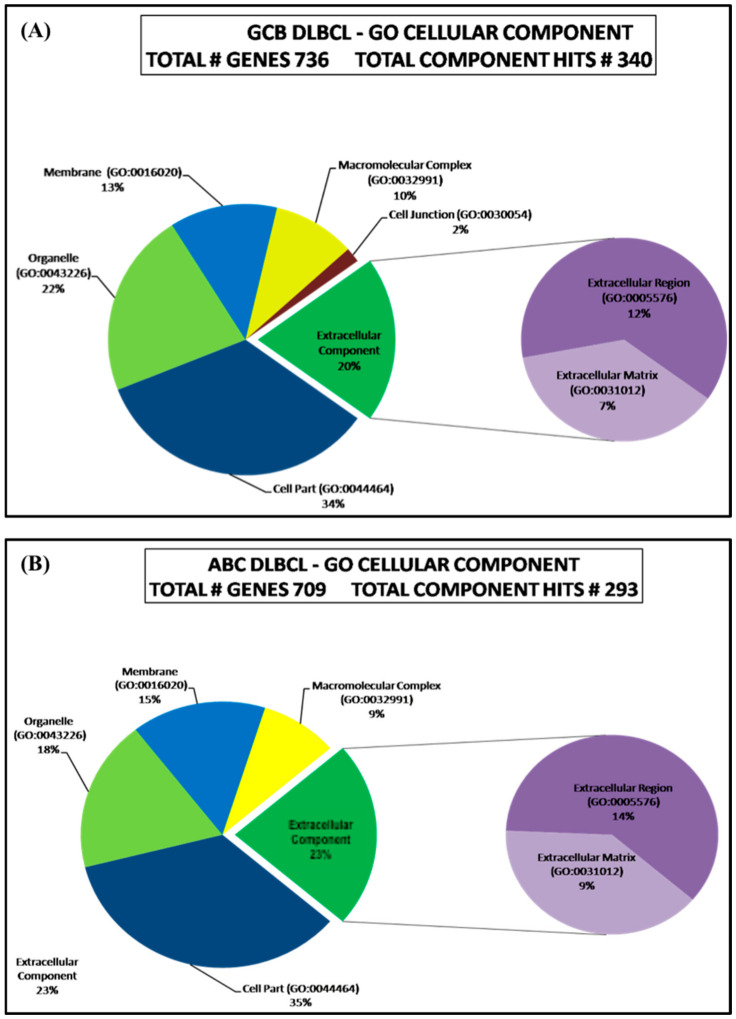
Mutated genes coding for extracellular components identified using Panther classification system: (**A**) 20% mutated extracellular components in GCB DLBCL, of which the extracellular region and extracellular matrix have 12% and 7% variants, respectively; (**B**) 23% mutated extracellular components in non-GCB DLBCL, of which the extracellular region and extracellular matrix have 14% and 9% variants, respectively. Abbreviations: GO, Gene Ontology; GCB, Germinal Center B-cell like; DLBCL, Diffuse Large B-Cell Lymphoma.

Genomic variants from WES data were analyzed for genomic variants coding for components of TME encompassing the extracellular matrix. SIFT and PolyPhen algorithms were implemented to identify variants with deleterious functional scores. We identified a total of 1445 novel variants of which 736 genomic variants were identified in GCB DLBCL cases, and, of note, 709 genomic variants were identified in non-GCB DLBCL cases. Gene ontology (GO) enrichment analysis gene coding for extracellular components was performed using the PANTHER classification system. Of the 736 variants, GO analysis identified 7% variants in the extracellular matrix, 12% variants from the extracellular region, and 20% variants of the extracellular component in GCB DLBCL cases. Similar analysis of non-GCB DLBCL cases identified 9% mutations in the extracellular matrix, 14% variants in the extracellular region, and 23% variants in the extracellular component (Figure 1).

Three common mutational variants were identified in GCB and non-GCB DLBCL cases including *ADAMTSL1*, *PRSS57*, *and FBN3*, and at chromosomal co-ordinates chr9:18680349, chr19:687142, and chr19:8154802, respectively. These common variants implicated in indel variant in *ADAMTSL1*(c.1176_1177delTGinsGT) and missense mutations in *PRSS57* (c.428C>T, p.Pro143Leu) and *FBN3* (c.6226C>T, p.Pro2076Ser). Of note, *SSPO* was mutated in both GCB and non-GCB DLBCL cases, but had different variants at different loci including chr7:149500578 (c.7979C>T), chr7:149484550 (c.3473G>A), and chr7:149513072 (c.10976_10976delT) (Table 1).

Mutation analysis in GCB DLBCL cases led to the identification of missense mutations in *CSN3*, i.e., c.46C>A, p.Pro16Thr; *SPARCL1*, i.e., c.565G>A, p.Gly189Arg; *COL4A2*, i.e., c.574G>T, p.Val192Phe; *COL5A3*, i.e., c.1219G>C, p.Gly407Arg; *IBSP*, i.e., c.922T>C, p.Tyr308His; *LRRC32*, i.e., c.1336C>T, p.Arg446Cys; *TMPRSS13*, i.e., c.205C>A, p.Ala69Thr; *COL13A1*, i.e., c.823C>T, p.Pro275Ser; *LAMC1*, i.e., c.3404G>T, p.Arg1135Leu; *USH2A*, i.e., c.15562A>G, p.Ser5188Gly; *LAMB1*, i.e., c.541C>T, p.Pro181Ser; *GAS6*, i.e., c.1714G>T, p.Gly572Cys; *STAB1*, i.e., c.5206G>C, p.Gly1736Arg, and an indel in *CPN2*, i.e., c.1525_1526delCAinsTG, p.Gln509Trp. In addition to these variants, *MUC5B*, *MUC2*, and *EGFLAM* had mutations specifically in GCB DLBCL cases. In non-GCB DLBCL cases, missense mutations were observed in *COL5A2*, i.e., c.2878G>C, p.Gly960Arg; *LTBP1*, i.e., c.4133T>C, p.Val1378Ala; *LAMA2*, i.e., c.1586G>A, p.Ser529Asn.

**Figure 2:**
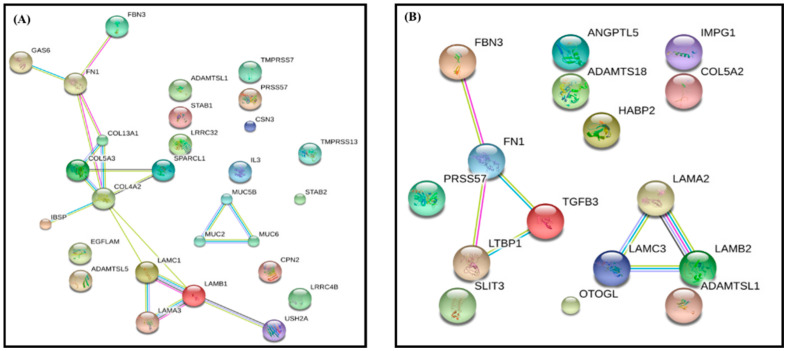
Protein–protein interaction amongst mutated stromal genes in (**A**) GCB DLBCL and (**B**) non-GCB DLBCL. Abbreviations: GCB, Germinal Center B-cell like; GO, DLBCL, Diffuse Large B-Cell Lymphoma.

**Figure 3:**
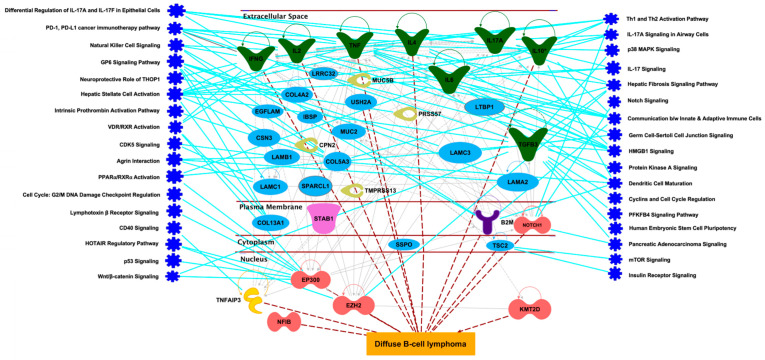
Ingenuity Pathway Analysis of stromal gene signatures in GCB DLBCL (left) and non-GCB DLBCL (right). Abbreviations: GCB, Germinal Center B-cell like; GO, DLBCL, Diffuse Large B-Cell Lymphoma.

STRING analysis was performed on mutated stromal genes in GCB and non-GCB DLBCL cases to identify plausible protein–protein interactions contributing significantly to extracellular components in GCB and non-GCB DLBCL phenotypes individually and variants shared across both GCB and non-GCB DLBCL phenotypes, respectively (Figure 2). Ingenuity Pathway Analysis of mutated stromal genes in GCB and non-GCB DLBCL cases from STRING analysis identified differential pathways in respective DLBCL subtypes (Figure 3).

### 3.2. Expression Profile of Mutated Stromal Genes

**Figure 4:**
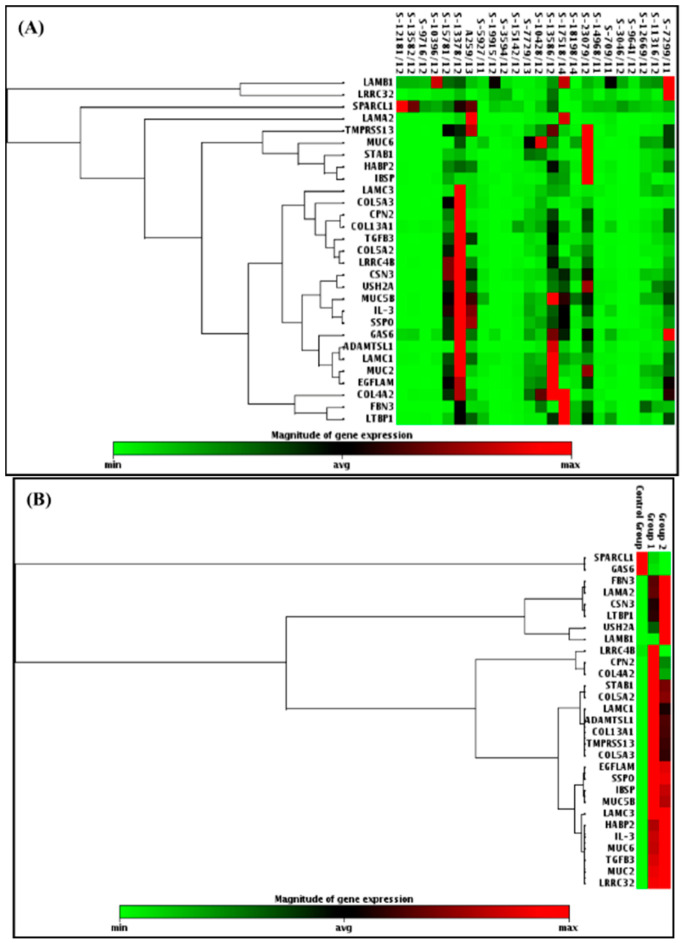
Relative expression clustergram of mutated stromal genes in DLBCL cases. (**A**) All DLBCL cases; (**B**) GCB DLBCL (Group 1) and non-GCB DLBCL (Group 2) compared to control group. Abbreviations: GCB, Germinal Center B-cell like; GO, DLBCL, Diffuse Large B-Cell Lymphoma.

The mutated stromal genes were then evaluated for genotype–phenotype correlation to study the effect of stromal mutations on expression analysis. Real-time PCR (RT-PCR) was performed to quantitatively evaluate the expression profile of 44 mutated stromal gene panels including genes coding for matrix metallo-proteinases, collagen, laminin, endothelial cells, receptors, structural protein, mucin, macrophages, and cytokines. *ADAMTSL1* showed a 2.5-fold downregulation in mutation-positive cases, whereas *SSPO* and *FBN3* expression in mutation-positive cases was 9-fold/4-fold downregulated, respectively, highlighting *ADAMTSL1*, *SSPO*, and *FBN3* as loss-of-function mutations. Of interest is *PRSS57*, which showed a 5.88-fold upregulation. In GCB DLBCL, the majority of the mutated genes lead to a gain-of-function effect in mutation-positive cases, including *LRRC32, EGFLAM, COL5A3, LAMB1. CPN2, COL13A1,* and *STAB1* with 7-, 16.62-, 4.98-, 4.87-, 2.77-, 10.63-, and 52-fold upregulation, whereas *USH2A* and *SPARCL1* were downregulated in mutation-positive cases (mean fold change: −17.94 and −11). However, no significant changes in gene expression were observed for *CSN3*, *COL4A2*, *GAS6, LAMC1, IBSP, MUC2*, *MUC5B,* and *MUC6* compared to controls. Interestingly, in non-GCB DLBCL significant gene expression downregulation was observed in *HABP2, LAMC3, COL5A2, LAMA2, LTBP1,* and *TGFB3* in mutation-positive cases (Figure 4).

### 3.3. OncoPrint of Gene Mutations Associated with the Stromal/Extracellular Matrix from Whole Exome Sequencing of DLBCL Patients from cBioPortal

**Figure 5:**
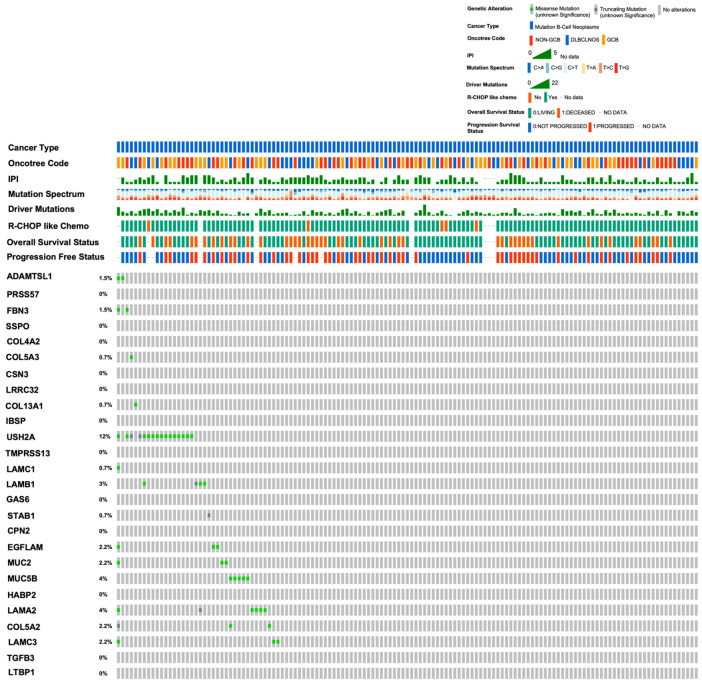
OncoPrint of mutated stromal genes from WES data of 135 DLBCL patients with tumor/matching normal samples pairs evaluated using cBioPortal (data accessible through dbGAP accession numbers phs000450 and GSE98588). OncoPrint demonstrates the genetic alteration type, oncotree code (GCB, non-GCB or DLBCL NOS), IPI score, mutation spectrum, R-CHOP status, overall survival, progression free survival, and frequency of mutated stromal genes in DLBCL cases.

In view of the results from our study, reporting the mutational profile of stromal genes for the first time and their potential role in DLBCL pathogenesis, we also analyzed the mutated stromal genes profile from WES data of matched tumor and normal patients (n = 135) using cBioPortal (data accessible through dbGAP accession numbers phs000450 and GSE98588) [11]. The OncoPrint analysis for the mutated stromal genes identified in our cohort were also reported in these patients (Figure 5). *USH2A* mutations were reported in 12% DLBCL patients, MUC5B and *LAMA2* mutations were present in 4%, followed by *LAMB1* mutations in 3% DLBCL cases. *EGFLAM*, *MUC2*, *COL5A2*, and *LAMC3* were mutated in 2.2% of DLBCL patients each and ≤1.5% mutations were observed in *ADAMTSL1*, *FBN3*, *COL5A3*, *COL13A1*, *LAMC1*, and *STAB1*. No mutations were reported in *TGFB3*, *LTBP1*, *HABP2*, *CPN2*, *GAS6*, *TMPRSS13*, *LRRC32*, *CSN3*, and *PRSS57*.

### 3.4. Stromal Gene Signature in GCB and Non-GCB DLBCL Predict SURVIVAL in DLBCL Patients

**Figure 6:**
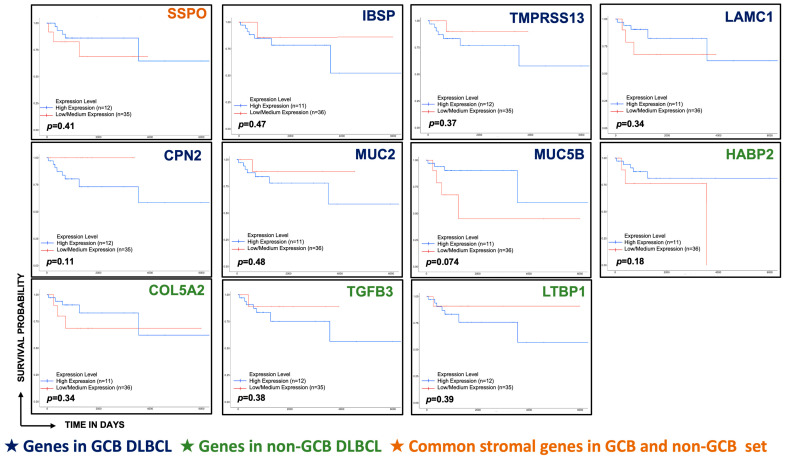
Survival analysis of stromal gene signatures in GCB and non-GCB DLBCL from TCGA—lymphoid neoplasm Diffuse Large B-Cell Lymphoma dataset.

To evaluate whether the identified stromal gene signatures showed any potential for predicting survival prognosis, we analyzed the Kaplan–Meier survival plots of stromal gene signatures from the TCGA ‘Lymphoid neoplasm Diffuse Large B-Cell Lymphoma’ dataset (Figure 6). Of the common genes, the high expression of *SSPO* was able to predict poor prognosis in DLBCL patients. Among the stromal gene signatures identified in the GCB DLBCL cases, six genes were able to predict survival from the TCGA- DLBCL dataset. Of the six genes, the high expression of *MUC5B* and *LAMC1* correlated with poor survival while the high expression of *MUC2*, *TMPRSS13*, *CPN2*, and *IBSP* predicted better survival in DLBCL patients. Similar analysis for the stromal gene signatures in the non-GCB phenotype illustrated four genes, i.e., *LTBP1*, *COL5A2*, *HABP2*, and *TGFB3*, could successfully predict prognosis in non-GCB DLBCL patients. Of these four genes, the low expression of *LTBP1* and *TGFB3*, whereas the high expression of *COL5A2* and *HABP2*, predicted poor prognosis. Based on TCGA survival analysis, we highlight the prognostic prediction of *MUC5B* to significantly predict survival in a small (n = 48) TCGA dataset, which can be extrapolated and studied in a larger DLBCL patient cohort for validation. To correlate the survival prediction profile with pathological data, we analyzed the histological images from the Human Protein Atlas (HPA) for these proteins in DLBCL patients compared to healthy tissue. Similar trends were visualized in HPA data from the same patients.

### 3.5. TME Modulates the Secreted Cytokine Profile of Malignant Cells

**Figure 7:**
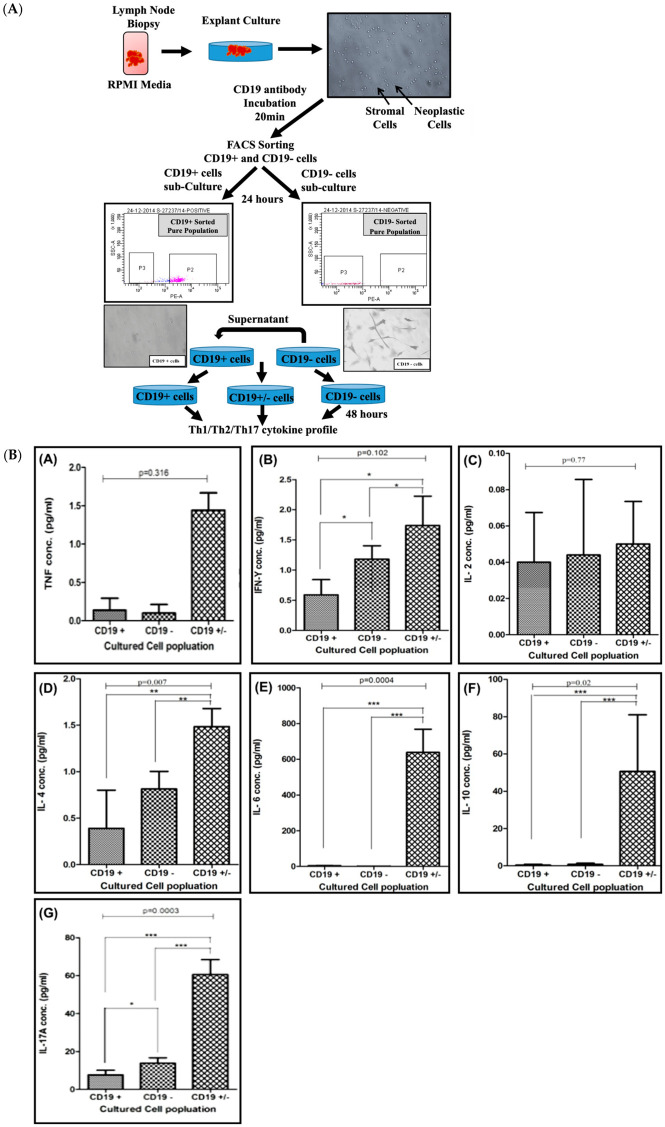
Tumor microenvironment modulates the secreted cytokine profile of malignant cells. (**A**) Primary cell culture of lymph node biopsy and sorting for CD19^+^/CD19^−^ cells using CD19 antibody in DLBCL cells. Gating strategy for sorting neoplastic cells (CD19^+^) and non-neoplastic cells (CD19^−^). Purity of sorted CD19^+^ and CD19^−^ cells was rechecked as illustrated in P2 (pink—CD19^+^ cells) and P3 (purple—CD19^−^ cells) (**B**) Serum cytokine of Th1/Th2/Th17 cytokines including IL-2, IL-4, IL-6, IL-10, IL-17A, TNF, and IFN-γ from CD19^+^, CD19^−^, and CD19 ^+^/^−^ culture supernatant, respectively. Data are illustrated as Mean ± SD with *p*-value (Mann–Whitney test * *p* < 0.05, ** *p* < 0.001, and *** *p* < 0.0001).

Cytokine analysis was performed on the culture supernatant from neoplastic cells cultured with TME culture supernatant and compared to stromal and malignant cells cultured individually using the BD^TM^ CBA Human Th1/Th2/Th17 Cytokine Kit. Th1/Th2/Th17 cytokine analysis illustrated elevated levels of Th17 (IL-17) and Th2 (IL-6, IL-4, and IL-10) in tumor cells cultured with TME culture supernatant IL-17 (*p* = 0.0003 ***), IL-6 (*p* = 0.0004 ***), IL-4 (*p* = 0.007 **), and IL-10 (*p* = 0.02 *) (Figure 7). Of the Th1 cytokines, TNF had comparatively high levels in CD19^+/−^ cells; however, it could not reach statistical significance (*p* = 0.316), and similar trends were observed for IFN-γ (*p* = 0.102) and IL-2 (*p* = 0.77).

## 4. Discussion

DLBCL pathogenesis owing to its heterogeneous presentation has still not been entirely understood. Besides the activated signaling pathway cascades and genetic mutations in neoplastic cells, the recent literature has documented the plausible role of potential cross-talk between tumor microenvironment and neoplastic cells during tumor progression in DLBCL. Furthermore, this is also reinforced by the clique highlighting bi-directional interactions between stromal and tumor cells eliciting a pathogenic role in the progression of lymphoid malignancies overall. There is limited literature om mutation profiling of stromal genes in DLBCL to date, which remains incompletely understood. This exploratory study provides first-hand evidence of mutations in genes associated with the stromal/extracellular matrix in DLBCL patients from an Indian cohort. In this study, we explored tumor stroma with an aim to ascertain novel stromal mutations coding for extracellular matrix components with deleterious PolyPhen and SIFT scores for the prediction of DLBCL prognosis in patients, and can be reciprocated as prospective stromal prognostic markers. We identified four stromal genes panel mutated across all DLBCL patients, including *PRSS57*, *FBN3*, and *ADAMTSL1*, whereas, interestingly, *SSPO* had mutations at distinct loci across different patients. Additionally, *CPN2*, *CSN3*, *COL5A3*, *COL4A2*, *COL13A1*, *LAMB1*, *LAMC1*, *IBSP*, *SPARCL1*, *TMPRSS13*, *USH2A*, *LRRC32*, *GAS6*, and *STAB1* were exclusively mutated in GCB DLBCL patients, whereas *TGFB3*, *HABP2*, *LAMC3*, *LAMA2*, *COL5A2*, and *LTBP1* were significantly mutated in non-GCB DLBCL patients. However, the heterozygous mutations only in *COL5A2, FBN3*, *ADAMTSL1*, *HABP2,* and *GAS6* could not be validated using Sanger sequencing, which may have been due to the low sensitivity of this platform to detect somatic mutations with <0.1% minor allele frequency (MAF) compared to WES with high coverage and sensitivity at the abovementioned chromosomal locations. Potentially, this could also be attributed to biopsy specimen, which is highly heterogenous and harbors a mixture of stromal and neoplastic cells at different frequencies at the center versus periphery.

Furthermore, the genotype–phenotype correlation was presented in this study for stromal gene variants by quantitative analysis. *FBN3* and *ADAMTSL1* were downregulated, whereas *SSPO* was 5.87-fold upregulated in mutation-positive DLBCL cases. Of the stromal variants mutated in GCB DLBCL cases, *COL5A3* was documented to be a predictor of survival by Rimsza et al. [26] (US Patent, US20150132297 A1), and its increased expression has also been associated with clear cell renal cell carcinoma progression [27]. In this study, we report a 4.97-fold upregulation of *COL5A3* variants in mutation-positive DLBCL cases, which is in accordance with the abovementioned studies highlighting *COL5A3* as a predictor of survival. *LRRC32* and *CPN2* were 7-fold and 2.7-fold upregulated, respectively, in mutation-positive patients as compared to their downregulation in mutation-negative cases. The *COL13A1* variant was upregulated and had a gain-of-function mutation in GCB DLBCL cases, which is in consensus with the findings from Tuomisto et al. [28], illustrating the altered expression of mutant collagen XIII’s correlation to the development of B-cell lymphoma [28]. Additionally, this is supported by the findings from Lenz et al. [15] demonstrating a high expression of stromal-1 marker: *COL13A1* is associated with a favorable prognosis in GCB DLBCL. *LAMB1* had a 4.87-fold higher expression and is in accordance with the findings from Alinezhad et al. [29], which reported *LAMB1* gene silencing that led to reduced tumor invasion. *STAB1* had a significant 52-fold high expression in GCB mutated cases, which may be attributed to *STAB1* being immunosuppressive in cancer [30,31]. *USH2A* and *SPARCL1* were 17.9-fold and 11-fold downregulated, while *EGFLAM* was reported to be 16.6-fold upregulated in GCB DLBCL cases. Though mutations in *USH2A* have been reported in splenic marginal zone lymphoma, thyroid cancers, and pediatric B-acute lymphoblastic leukemia, there is limited literature deciphering the role of *USH2A* [32,33,34,35]. Of interest is *SPARCL1*, with contrasting literature documenting its downregulation, which inhibits metastatic cancer progression in prostate cancer, renal cancer, and gastric adenocarcinoma, and is a good predictor of prognosis [36,37,38]. In a study by Hurley et al., it was associated with reduced survival in mutation-positive cases [32].

In the variants identified in non-GCB-DLBCL, a substantial downregulation was observed across the variants quantitated using gene expression analysis. Of these, *COL5A2*, *TGFB3*, *HABP2*, *LAMA2*, *LTBP1*, and *LAMC3* had 16.79-, 12.6-, 2.97-, 29.6-, 4.3-, and 4.3-fold downregulation of gene expression, respectively. Of these genes, *COL5A2* was upregulated in mutation-negative DLBCL cases, which is in concordance with the findings reporting *COL5A2*’s elevated expression in cancers [39,40]. *HABP2* has been reported to be recurrently mutated in lung and thyroid cancer and also have an elevated expression in the kidney, liver, breast, and brain [41,42,43]. In a study by Lu et al., the downregulation of *LAMA2* was reported to mediate the endothelial-to-mesenchymal transition in cancer, which is in agreement with our data reporting *LAMA2*’s downregulation in DLBCL patients [44]. *LTBP1* on the other hand plays a dual role in different cancers, where its variable expression profile has been reported in T-cell lymphomas, breast carcinomas, and other malignancies [45,46]. *LAMC3* has been documented as a tumor suppressor gene in breast cancer and colorectal cancer, which is in sync with the observations from our study reporting the downregulation of *LAMC3* in non-GCB DLBCL cases [45,46].

Hence, in view of these findings, the stromal variants reported in this study using WES, and validated using the Sanger sequencing platform, illustrate potential stroma variants in DLBCL patients, which are promising mutated stromal markers for stratifying DLBCL patients prognostically.

In addition to identifying stromal variants coding for TME, we also evaluated the effect of pathogenic mutations on the cytokine profile, which was studied through primary cell cultures of DLBCL cases. We attempted to evaluate potential humoral biomarkers from TME in an in vitro primary cell culture study on a DLBCL lymph node biopsy, evaluating the cross-talk between malignant cells and the TME. This unique in vitro study design is the first of this kind to evaluate and document the role of TME in DLBCL progression. Additionally, direct co-culture experiments may be performed for additional biological insights and the recapitulation of in vivo TME, however this was beyond the scope of the present experimental design. The culture supernatant evaluated for serum cytokine profiling illustrated significant upregulation of Th2/Th17 cytokines in culture supernatant with malignant cells and TME compared to CD19^+^ malignant cells and CD19^−^ non-malignant cells alone, thereby advocating the role of TME interactions with direct proportionality to tumor progression. The observations from our study are in agreement with the results from Mori et al. [47], which concluded Th1/Th2 balance is polarized towards Th1 in DLBCL patients with complete remission, and towards Th2 in untreated patients. In our study, we observed decreased serum IL-2 levels in DLBCL cases in comparison to the control group. Similar findings were reported by Fatemeh Saberi Hosnijeh et al. [48] who documented an inverse association between NHL occurrence and IL-2 cytokine profile, thus indicating proportionality between the downregulation of Th1 cytokines and increased risk of relapse. Additionally, in our study, TNF (tumor necrosis factor), a cytokine playing a central role in inflammatory and immune responses [49], had a relatively high expression in DLBCL patients, though difference could not achieve statistically significance. Similar results were reported by Zhu et al. [50], where TNF-α was not found to predict a clinical staging of the disease. Hence, the elevated TNF-α levels may be attributed to its secretion by reactive lymphoid cells or neoplastic cells. Interleukin 6 (IL-6), a multifunctional cytokine, is actively involved in regulating acute phase response, T-cell proliferation, differentiation of monocytes to macrophages, and Th2 cytokine production [51,52].

It also acts as an important tumor-promoting factor across different cancers including lymphoma, solid tumors, and myeloma [53,54]. In this study, we observed a significant elevation of IL-6 serum levels (*p* ≤ 0.0001) in DLBCL patients. These observations are in consensus with previous studies reporting 35–78% elevated IL-6 levels in different malignancies [55,56,57,58,59,60,61,62] with a positive correlation of IL-6 levels with advanced-stage, elevated LDH, B symptoms, and high-risk IPI. The serum IL-10 levels have been reported to be significantly elevated in NHL patients as compared to NHL patients in remission and healthy individuals [63]. In the current study, we observed significantly increased IL-10 levels in DLBCL patients in comparison to healthy controls (*p* = 0.0004), which is in accordance with previous reports documenting elevated IL-10 levels observed in 65–72% of DLBCL patients [57,58,61,64]. Furthermore, in a study by Lan et al., cytokine polymorphisms in the Th1/Th2 pathway illustrated that SNPs in Th2 cytokine might be correlated with NHL risk [65], highlighting the importance of maintaining a Th1/Th2 balance shift, which may lead to lymphomagenesis. Further functional and mechanistic studies with a larger sample size would establish the causality of tumor–stroma interactions.

In summary, this study highlights the importance of stromal gene signatures as prognostic markers in DLBCL. We propose differential stromal gene markers for GCB and non-GCB DLBCL subtypes, which have prognostic value. However, the mutations in genes associated with the stromal/extracellular matrix would need further exploration to replicate them as prognostic and diagnostic makers in a clinical setting. The cross-talk between the lymphoma and stromal cells in TME is important for lymphomagenesis, wherein cytokines could evolve as potential prognostic biomarkers.

## 5. Conclusions

Taken together, DLBCL immunohistochemical subtypes have limited prognostic significance in the clinical cohort of patients. Though the International Prognostic Index (IPI) successfully predicts prognosis in DLBCL patients (EFS and OS), variable treatment response to conventional chemotherapy was observed amongst similar IPI groups. Given the biological heterogeneity of DLBCL and variable therapeutic response, TME is helpful for understanding disease progression and therapeutic resistance, which remains incompletely understood. This exploratory study identifies novel mutations in genes associated with the stromal/extracellular matrix, with significant genotype–phenotype correlations as potential prognostic markers in DLBCL. Th2 and Th17 polarization in tumor cells from in vitro primary cell culture and serum cytokine profiling could evolve as potential prognostic biomarkers. The findings from this study present a step forward in understanding TME in DLBCL pathogenesis with novel mutational targets, which can evolve as potential targets for effective therapeutic management in DLBCL patients.

## Figures and Tables

**Table 1:** Mutations identified in tumor pathway and stromal genes in GCB and non-GCB DLBCL cases, with mutations specific to GCB DLBCL and non-GCB DLBCL, respectively, and mutations common in both GCB and non-GCB DLBCL.

Gene	Locus	Ref	Genotype	Type	Transcript	Nucleic Acid Change	Amino Acid	Variant Effect	dbSNP/ COSMIC	Known/Novel	Sift	Polyphen	*p*-Value
Tumor Pathway Mutations													
GCB DLBCL													
*EZH2*	chr7:148508727	T	T/A	SNV	NM_004456.4	c.1937A>T	p.Tyr646Phe	Missense	37029:220730	Known	0	1	0.00001
*EP300*	chr22:41566522	T	T/G	SNV	NM_001429.3	c.4399T>G	p.Tyr1467Asp	Missense	rs200897987	Known	0	1	0.00001
*NOTCH1*	chr9:139390648	CAG	CAG/C	INDEL	NM_017617.3	c.7541_7542delCT	p.Pro2514fs	Frameshift deletion	12774:1292819	Known	0.01	0.989	0.00001
*KMT2D*	chr12:49416500	G	G/C	SNV	NM_003482.3	c.16211C>G	p.Ser5404Cys	Missense	221012:221013	Known	0	1	0.01135
*TSC2*	chr16:2122311	A	A/AC	INDEL	NM_000548.3	c.2167_2168insC	p.Ile723fs	Frameshift insertion		Known	0.03	0.889	0.00084
*B2M*	chr15:45003779	T	T/A	SNV	NM_032504.1	c.35T>A	p.Leu12Gln	Missense	221273:1373120	Known	0.16	1	0.00001
NON-GCB DLCBL													
*TNFAIP3*	chr6:138195991	A	A/G	SNV	NM_001270507.1	c.305A>G	p.Asn102Ser	Missense	rs146534657, 36249	Known	0	1	0.00001
*NFIB*	chr9:14307354	G	G/A	SNV	NM_001190737.1	c.196C>T	p.Pro66Ser	Missense	rs140030018/ 1581308:1581309	Known	0	0.948	0.00001
Stromal Mutations													
Common Mutations													
*ADAMTSL1*	chr9:18680349	TG	GT/GT	MNV	NM_001040272.5	c.1176_1177delTGinsGT	p.Cys392_Gly393delinsTrpTrp	Missense	rs199787607	Known	0	1.0	0.00001
*PRSS57*	chr19:687142	G	A/A	SNV	NM_214710.3	c.428C>T	p.Pro143Leu	Missense	rs8102982	Known	0.08	1.0	0.00001
*SSPO*	chr7:149484550	G	G/A	SNV	NM_198455.2	c.3473G>A	p.Arg1158Gln	Missense	rs188422566	Known	0.06	0.476	0.00001
	chr7:149500578	C	C/T	SNV	NM_198455.2	c.7979C>T	p.Pro2660Leu	Missense	rs374606341	Known	0.0	1.0	0.00001
	chr7:149513072	GTTTC	GTTC/GTTTT	INDEL	NM_198455.2	c.10976_10976delT	p.Ser3660fs	Frameshift deletion	rs370886797	Known	0.02	0.997	0.00001
*FBN3*	chr19:8154802	G	G/A	SNV	NM_032447.3	c.6226C>T	p.Pro2076Ser	Missense	-	Novel	0.08	1.0	0.00001
GCB DLBCL													
*COL4A2*	chr13:111082772	G	G/T	SNV	NM_001846.2	c.574G>T	p.Val192Phe	Missense	rs62621885	Known	0.12	0.846	0.00001
*CSN3*	chr4:71110582	C	C/A	SNV	NM_005212.2	c.46C>A	p.Pro16Thr	Missense	rs374280097	Known	0	0.999	0.00001
*SPARCL1*	chr4:88415387	C	C/T	SNV	NM_004684.4	c.565G>A	p.Gly189Arg	Missense	rs201923671	Known	0.01	0.028	0.02265
*LRRC32*	chr11:76371301	G	G/A	SNV	NM_005512.2	c.1336C>T	p.Arg446Cys	Missense	rs148695421	Known	0	0.997	0.00001
*IBSP*	chr4:88733030	T	T/C	SNV	NM_004967.3	c.922T>C	p.Tyr308His	Missense	rs370483467	Known	0	1	0.00001
*USH2A*	chr1:215799170	T	T/C	SNV	NM_206933.2	c.15562A>G	p.Ser5188Gly	Missense	rs58257972	Known	0	0.911	0.00001
*LAMC1*	chr1:183099602	G	G/T	SNV	NM_002293.3	c.3404G>T	p.Arg1135Leu	Missense	rs150090402	Known	0.15	0.532	0.00001
*CPN2*	chr3:194061906	TG	TG/CA	MNV	NM_001080513.2	c.1525_1526delCAinsTG	p.Gln509Trp	Indel	rs4974538, rs4974539	Known	0	0.996	0.00001
*STAB1*	chr3:52553551	G	G/C	SNV	NM_015136.2	c.5206G>C	p.Gly1736Arg	Missense	rs143894786	Known	0	1	0.00001
*EGFLAM*	chr5:38370532	G	G/A	SNV	NM_001205301.1	c.680G>A	p.Arg227His	Missense	rs199795131	Known	0.07	0.988	0.00001
*MUC5B*	chr11:1260726	CGGGGCA	CGGGCAG/CGGGGCAG	MNV, INDEL	NM_002458.2	c.3517_3519delGCAinsCAG, c.3519_3520insG	p.Ala1173Gln, p.Pro1174fs	Missense, Frameshift Insertion	rs371432801	Known	0	0.998	0.00001
*GAS6*	chr13:114525099	C	C/A	SNV	NM_000820.2	c.1714G>T	p.Gly572Cys	Missense	-	Novel	0.03	0.999	0.00001
*LAMB1*	chr7:107626691	G	G/A	SNV	NM_002291.2	c.541C>T	p.Pro181Ser	Missense	-	Novel	0.07	0.967	0.00001
*TMPRSS13*	chr11:117789370	C	T/T	SNV	NM_001077263.2	c.205C>A	p. Ala69Thr	Missense	-	Novel	0.08	0.97	0.00001
*COL5A3*	chr19:10108091	C	CG/G	SNV, INDEL	NM_015719.3	c.1219G>C, c.1218_1219insC	p.Gly407Arg, p.Gly407fs	Missense, Frameshift insertion	-	Novel	0	1	0.00001
*COL13A1*	chr10:71662546	C	C/T	SNV	NM_001130103.1	c.823C>T	p.Pro275Ser	Missense	-	Novel	0	0.858	0.00001
*MUC2*	chr11:1094754	A	A/C	SNV	NM_002457.3	c.5830A>C	p.Thr1944Pro	Missense	-	Novel	0.07	0.982	0.00001
*MUC6*	chr11:1031714	T	T/C	SNV	NM_005961.2	c.376A>G	p.Thr126Ala	Missense	-	Novel	0.31	0.757	0.00001
*LRRC4B*	chr19:51021625	C	C/G	SNV	NM_001080457.1	c.1345G>C	p.Ala449Pro	Missense	-	Novel	0	1	0.00001
NON_GCB DLBCL													
*LAMA2*	chr6:129511468	G	G/A	SNV	NM_000426.3	c.1586G>A	p.Ser529Asn	Missense	rs370691060	Known	0.01	0.183	0.00001
*LTBP1*	chr2:33585796	T	C/G	SNV	NM_206943.2	c.4133T>C, c.4133T>G	p.Val1378Ala, p.Val1378Gly	Missense	rs4422143	Known	0.05	0	0.00001
*HABP2*	chr10:115340418	G	C/GC	SNV, INDEL	NM_004132.3	c.805G>C, c.805_806insC	p.Glu269Gln, p.Glu269fs	Missense	-	Novel	0.02	0.999	0.00001
*COL5A2*	chr2:189916099	C	C/G	SNV	NM_000393.3	c.2878G>C	p. Gly960Arg	Missense	-	Novel	0	1	0.00001
*LAMC3*	chr9:133901690	C	C/T	SNV	NM_006059.3	c.392C>T	p.Thr131Met	Missense	-	Novel	0	1	0.00001
*TGFB3*	chr14:76425616	GC	GC/CG	MNV	NM_003239.2	c.1152_1153delGCinsCG	p.Glu384_Pro385delinsAspAla	Indel	-	Novel	0	0.999	0.00001

*HABP2*, i.e., c.805G>C, p.Glu269Gln; *LAMC3*, i.e., c.392C>T, p.Thr131Met, whereas an indel was observed in *TGFB3*, i.e., c.1152_1153delGCinsCG, p.Glu384_Pro385delinsAspAla) (Table 1). The panels of stromal mutations identified using WES underwent validation through the Sanger sequencing platform (Supplementary Figure S3).

## Data Availability

The raw sequence data generated from this study have been deposited in the National Center for Biotechnology Information (NCBI) Sequence Read Archive (SRA) and are available under SRA data accession: SRP314394; BioProject Accession: PRJNA721234; BioSample Accession: SAMN18711219–SAMN18711226.

## Supplementary Materials

The following supporting information can be downloaded at: https://www.mdpi.com/article/10.3390/cancers18111697/s1, Table S1: PCR primer sequences used for PCR amplification and Sanger sequencing. Table S2: Real-time PCR primer sequences used for qPCR. Table S3: RNA concentration and quality of DLBCL samples subjected to PCR array (50 ng of total RNA was used for the reverse transcription of each sample). Figure S1: The photomicrographs show the immunohistochemical staining profile used for the classification of Diffuse Large B-Cell Lymphoma cases into Germinal Center B-cell like (GCB) and activated B-cell (ABC) subtypes using Han’s algorithm. H&E staining in DLBCL cases at (A) ×200 and (B) ×400 showing large nuclei. (C,D) DLBCL cases were semi-quantitated for BCL-6, CD10, and MUM1 protein expression with a cut-off staining score of ≥30%. (C) The GCB phenotype was diagnosed if there was CD10 (membranous) and BCL-6 (nuclear) positivity and a lack of MUM1 expression (top panel). (D) Alternatively, the non-GCB subtype lacks CD10 and BCL-6 expression but exhibits MUM1 positivity (bottom panel) (original magnifications: X100 [low] and X400 [high]). Abbreviations: GCB, Germinal Center B-cell like; ABC, Activated B-cell-like; GO, Gene Ontology; DLBCL, Diffuse Large B-Cell Lymphoma; BCL6, B-cell Lymphoma 6; CD10, Cluster of Differentiation 10; MUM1, Multiple Myeloma Oncogene 1. Figure S2: Real-time plate customized to evaluate the expression of forty-four genes (ACTB as housekeeping genes), genomic DNA contamination control, and non-template control (RTC). Figure S3: (A–W) Sanger validation of stromal mutations identified from whole exome sequencing data.
