# Supplementary material for: Pathogenic Mutations in the Tumor Microenvironment Drive Tumor Progression in Diffuse Large B-Cell Lymphoma Through Tumor–Stroma Cross-Talk
Source: Cancers (Basel). 2026 May 22;18(11):1697. doi: 10.3390/cancers18111697 (PMC13255756; doi:10.3390/cancers18111697)
Supplement: Supplementary file 1 [file cancers-18-01697-s001.zip › cancers-4298182-supplementary.pdf]

Article

Pathogenic mutations in the tumor microenvironment drive tumor progression in diffuse large B-cell lymphoma through tumor–stroma cross-talk

Vaishali Aggarwal [1,2,3], Radhika Srinivasan [4], Amanjit Bal [1], Pankaj Malhotra [5], Subhash Varma [5,6] and Ashim Das [1,7,*]

[1]Molecular Biology Laboratory, Department of Histopathology, Post Graduate Institute of Medical Education and Research (PGIMER), Chandigarh, 160012, India; vaishali.pgi@gmail.com (V.A.); docaman5@hotmail.com (A.B.); ashim126@gmail.com (A.D.)

[2]Department of Immunology, University of Pittsburgh School of Medicine, Pittsburgh, PA, 15213, USA; vaa30@pitt.edu (V.A.)

[3]Tumor Microenvironment Center, UPMC Hillman Cancer Center, Pittsburgh, PA, 15213, USA; vaa30@pitt.edu (V.A.)

[4]Department of Cytology and Gynaecological Pathology, PGIMER, Chandigarh, 160012, India; drsradhika@gmail.com (R.S.)

[5]Department of Clinical Hematology and Medical Oncology, PGIMER, Chandigarh, 160012, India; malhotrapankaj@hotmail.com (P.M.)

[6]Internal Medicine, Fortis Hospital, Mohali, India; suvarma@gmail.com (S.V.)

[7]Department of Hepatopathology, Kalinga Institute of Medical Sciences (KIMS), Bhubaneswar.

* Correspondence: ashim126@gmail.com; Tel.: +91 9872223744

Supplementary Table S1: PCR primer sequences used for PCR amplification and Sanger sequencing.

GENE	PRIMER	PRIMER SEQUENCE	PRODUCT LENGTH (bp)	Tm (°C)
**ADAMTSL1**	F	5′- TAGGTTACAGACGGCACACA-3′	546	63
	R	5′-TGATGATGAGAGGGAAGAGTGG -3′		
**COL5A3**	F	5′-AAAAGAAGAGGGGTCCGAGAGTA-3′	394	59.8
	R	5′ATGGGAGACAGAGAACAGAAGAGG-3′		
**CPN2**	F	5′-ATCGGCTCCTGAACATCCA-3′	649	58.5
	R	5′- GCTTTTGAGGGTGCTTTGC-3′		
**CSN3**	F	5′-TTCACATCGGCTAAATCTACCT-3′	750	63
	R	5′-TGGAAAGGGGTCAGTCAAT-3′		
**FBN3**	F	5′-AGACCCCTGCGAACTGTGT-3′	427	59.8
	R	5′-ACCACACAATCGCCAGAG-3′		
**COL4A2**	F	5′-ATGGGAGGGGTAATGAAGG-3′	563	56.8
	R	5′-GCAGCGGGAGAATGTTTT-3′		
**MUC6**	F	5′-CAGCACAAACAAAACACCTACCTC-3′	476	58.5
	R	5′-TGGTCAGCGTCATTGTTG-3′		
**LRRC4B**	F	5′-ACCAGTCGCATTTCACCT-3′	405	57.5
	R	5′-CCGTGGTGAAGTAGGTGTAGC-3′		
**PRSS57**	F	5′-TTCACAAGGGCAGGACCA-3′	612	58.5
	R	5′-ACTACCCTCTACTGAATACTGCTTG-3′		
**SPARCL1**	F	5′-CAGCAGAACAGGGCAAGAGT-3′	455	58.5
	R	5′-TTCCTCCTGCTTGCTGTTAG-3′		
**COL5A2**	F	5′-TGCGAGGCTTGGACATCT-3′	298	57.5
	R	5′-AAGGTGGTCTGGAACGGATA-3′		
**COL13A1**	F	5′-CATCTTGGCGTTCTTCTCAC-3′	482	57.5
	R	5′-AGTGTGATGGGGATGAATAGCC-3′		
**HABP2**	F	5′-ATGAGACTTGGGACCGCTTT-3′	550	58.5
	R	5′-GGACTACCTGACCTAAACCCACA-3′		
**LAMC3**	F	5′-CCAGAAGAGGGACAGCAAATAC-3′	606	57.5
	R	5′-TGGCACATCAGACCCTCCTA-3′		
**TGFB3**	F	5′-CAACTACTCACTCACCCAAACCA-3′	688	58.4
	R	5′-AGTCAGGCAGTGGTGGTTCTCT-3′		
**LAMA2**	F	5′-CCATCCAATAATCTTTCTCACCAG-3′	644	58.4
	R	5′-GCACCCTTGGAGTTGAAA-3′		
**LTBP1**	F	5′-CACTCTTTCCCCCATTGC-3′	360	58.5
	R	5′-GGCTGTGGGAATGAGTGTTATC-3′		
**STAB1**	F	5′-TTCCGAGCACCCCACCTAT-3′	719	59.4
	R	5′-CAGCAAAGATGTGGCAGTC-3′		
**GAS6**	F	5′-CATCCACAGTCAGCGAGCA-3′	501	58.5
	R	5′-CTTCCGCATTCACTACCAGA-3′		
**LAMC1**	F	5′-GGTTGGAGGTGTGATGAGTG-3′	620	59.2
	R	5′-AAATAACCCAGATGTCCCTTG-3′		
**USH2A**	F	5′-CTGTTTTAGACCCCACCAT-3′	304	56.8
	R	5′-GAGTGATAACCCAGGAGGAAAT-3′		
**IBSP**	F	5′-CCTACAACCCCACCACAAGTCTAT-3′	523	55.4
	R	5′-GCTACAACCTCAAACCATTCAA-3′		
**LAMB1**	F	5′-GCAGTTCAAGTTTGTGGCA-3′	311	56.8
	R	5′-AGAAGCAAACAAACCTCTCCT-3′		
**LRRC32**	F	5′-AACTGCTTGCGGACCTTTG-3′	506	58.4
	R	5′-TTGAGGCAGATGAAGCAG-3′		

**MUC5B**	F	5′-CCGCACACAGACATCCAA-3′	548	63
	R	5′-GCAGTTTACGGGAGCCTACA-3′		
**TMPRSS13**	F	5′-GGGACAGCAGGTTCTTTGA-3′	654	59.2
	R	5′-TCTCGTGGCTTCCCTACA-3′		

Supplementary Table S2: Real-time PCR primer sequences used for qPCR.

GENE	PRIMER	PRIMER SEQUENCE	PRODUCT LENGTH (bp)	Tm (°C)
**ADAMTSL1**	F	5′-CCTGCCTATTGACGAGTGTGA-3′	107	58
	R	5′- CATCTGTCTCGTCTGGGTTGA-3′		
**COL5A3**	F	5′-TGCCTGATGGGGAATACTGG-3′	109	58
	R	5′-TTGTCGGGATAGAGGCAGG-3′		
**CPN2**	F	5′-TGGGCAGCAACAACCTTAC-3′	200	58
	R	5′-CAGCCAGTTGAAGAGGTAGG-3′		
**CSN3**	F	5′-CTCCTGCCACTGAACCAAC-3′	123	58
	R	5′-GGTGTTTTTATGCCGTAGGTGG-3		
**FBN3**	F	5′-GAGTTTCTGCTTCACCCGTT-3′	72	58
	R	5′-CGGGTCTTGGTGGTGTTGA-3′		
**COL4A2**	F	5′-GTTTGATGTGCCGTGTGG-3′	172	58
	R	5′-TCCCCTTTCACCCTTGTCT-3′		
**MUC6**	F	5′-GTGAAGACCTGCTCCAACC-3′	107	58
	R	5′-CAGGTGTGGTTATTGGAGAGG-3′		
**LRRC4B**	F	5′-CGGCTACACCTACTTCACCAC-3′	175	58
	R	5′-TGCCGTGGTAGAAGACGAG-3′		
**SPARCL1**	F	5′-TTGGCTCCTGGTGTTAGTTCC-3′	131	58
	R	5′-CCTTGGCTATGTTTACTGCTCC-3′		
**COL5A2**	F	5′- TGTGGTTCTCAAAGGGGCAA-3′	112	58
	R	5′- CTTGCCCACATTTCCATTCC-3′		
**COL13A1**	F	5′- GATGACAGGACCAACGGGA-3′	151	58
	R	5′- GCAGGGGGCATCTAATCCA-3′		
**HABP2**	F	5′- TTATTGGAAAGCCTGGACCC-3′	89	58
	R	5′- GTGCTACTGGTGTTCTCTTCCT-3′		
**LAMC3**	F	5′- CTGGGTTTCTTCGGCTTCTC-3′	94	58
	R	5′- ATGTGCCGTTCTCGTGG-3′		
**TGFB3**	F	5′- GCTACTATGCCAACTTCTGCTC-3′	103	58
	R	5′- GCTTCAGGGTTCAGAGTGTTG-3′		
**LAMA2**	F	5′- GGTTCCAGGGCATTTGTGTG-3′	122	58
	R	5′- TAGATGCTGGGTTTGGGCTT-3′		
**LTBP1**	F	5′- GTCAGAATGGAGGGATGTGT-3′	172	58
	R	5′- GTATGCTTTGCTGCTTGCTC-3′		
**STAB1**	F	5′- GGGATGCTATTGGGCTATGC-3′	103	58
	R	5′- CATTGACAGGGACGAAGAGTG-3′		
**GAS6**	F	5′-GGAGCGAGGACTGTATCATCT-3′	128	58
	R	5′-CTTCTCCGTTCAGCCAGTTC-3′		
**LAMC1**	F	5′-CGGCTGGTAAAGGATAAGGTTG-3′	94	58
	R	5′-TCACCATCTCATCCCCAGTT-3′		
**USH2A**	F	5′-AAGGTTTTGTGGGCTGTCTC-3′	80	58
	R	5′-CCAATCCAGAGGTTCCCAAA-3′		
**IBSP**	F	5′-AACCACTTCCCCACCTTTTG-3′	121	58
	R	5′-AGGTTCCCCGTTCTCACTT-3′		
**LAMB1**	F	5′-CGTTTTACTACCAGCACCCA-3′	137	58

	R	5′-GCCAGCAATGAGACCAGTAGA-3′		
**LRRC32**	F	5′-AAGGTCTCGTGCCAGGTT-3′	140	58
	R	5′-AGGTGACGAAGTGCTGTGTAG-3′		
**MUC5B**	F	5′-AGAGCGGGGACTACATCAA-3′	108	58
	R	5′-AGGTCTGGTTGGCGTATTTG-3′		
**TMPRSS13**	F	5′-GTGGTTTCGCTCATCATCCT-3′	159	58
	R	5′-CCAGTCAAACCTCACGCA-3′		
**CAV1**	F	5′-GCGACCCTAAACACCTCAA-3′	88	58
	R	5′-CCGTCAAAACTGTGTGTCC-3′		
**MMP9**	F	5′-GCACCACCACAACATCACC-3′	79	58
	R	5′-GGCAAAGGCGTCGTCAATC-3′		
**ERG**	F	5′-CCCGTGACATCTTCCAGTTT-3′	121	58
	R	5′-AGTAAGTGCCCAGATGAGAAGG-3′		
**THBS1**	F	5′-ACGCCATCAGGGTAAAGAAC-3′	129	58
	R	5′-TAGTCATCGTCCCTTTCGGT-3′		
**SSPO**	F	5′-CCTTGTGACGGTGTTCTGG-3′	98	58
	R	5′-GTCTTGGCTGTGGAATGGAA-3′		
**CD14**	F	5′-TAGACCTCAGCCACAACTCG-3′	100	58
	R	5′-CCAGCGAACGACAGATTGAG-3′		
**PECAM1**	F	5′-AAGTAAGGTGGTGGAGTCTGG-3′	184	58
	R	5′-ATACTCTCCCTCCTGTTCCTTG-3′		
**MUC2**	F	5′-TGCGACTACTACAACCCTCC-3′	121	58
	R	5′-CCAGGTAGGACACGGAGATG-3′		
**EGFLAM**	F	5′-AACCGTGGATGACTATGGAGC-3′	95	58
	R	5′-CCTTCATTCCACCCACATACAG-3′		
**KDR**	F	5′-CACTGTCATCCTTACCAATCCC-3′	82	58
	R	5′-AATCTGGGGTGGGACATACA-3′		
**FN1**	F	5′-AGCAGACCCAGCTTAGAGTT-3′	134	58
	R	5′-GCAGAAGTGTTTGGGTGACT-3′		
**IL-6**	F	5′-GACAGCCACTCACCTCTTC-3′	120	58
	R	5′-CCTCTTTGCTGCTTTCACAC-3′		
**IL-17A**	F	5′-TCAACCCGATTGTCCACCAT-3′	136	58
	R	5′-GAGTTTAGTCCGAAATGAGGCTG-3′		
**IFN-γ**	F	5′-GACCAGAGCATCCAAAAGAGTG-3′	178	58
	R	5′-GCGACAGTTCAGCCATCA-3′		
**TNF**	F	5′-ATGAGCACTGAAAGCATGATCC-3′	217	58
	R	5′-GAGGGCTGATTAGAGAGAGGTC-3′		
**IL-4**	F	5′-CAGTTCCACAGGCACAAGCA-3′	107	58
	R	5′-CTCTGGTTGGCTTCCTTCACA-3′		
**IL-3**	F	5′-GCGATCTTTTGAGTCCAACG-3′	126	58
	R	5′-GCTTCTGGTCCTGGAATGTG-3′		
**IL-2**	F	5′- CCTGAGCAGGATGGAGAATTACA-3′	145	58
	R	5′- TCCAGAACATGCCGCAGAG-3′		
**IL-10**	F	5′-GCCTAACATGCTTCGAGA-3	206	58
	R	5′-TGATGTCTGGGTCTTGGTTC-3′		
**B-ACTIN**	F	5′ AGAGCTACGAGCTGCCTGAC-3′	184	58
	R	5′-AGCACTGTGTTGGCGTACAG-3′		

Supplementary Table S3: RNA concentration and quality of DLBCL samples subjected to PCR array (50ng of total RNA was used for the reverse transcription of each sample).

S.No.	Sample No.	RNA concentration (ng/	A260/A280	A260/A230
1	S-7299/11	673	2.1	1.8
2	S-11316/12	689	2.2	1.9
3	S-12669/12	1568	2.0	1.7
4	S-9641/12	1286	2.1	1.9
5	S-3046/12	985	2.0	2.0
6	S-709/11	786	2.0	1.9
7	S-14968/11	1450	2.0	1.8
8	S-23079/12	868	1.9	1.9
9	S-18198/14	794	1.9	1.8
10	S-17518/14	569	2.1	1.9
11	S-13586/12	1234	1.9	2.0
12	S-10428/12	1197	1.9	1.9
13	S-7729/13	861	2.0	1.9
14	S-15142/12	974	2.0	1.8
15	S-3594/12	852	2.1	1.8
16	S-19915/12	1581	2.2	1.7
17	S-5927/11	986	2.2	1.9
18	A259/13	1055	2.0	2.0
19	S-13378/12	796	2.1	2.0
20	S-15781/12	862	2.1	2.0
21	S-10396/12	1639	2.0	1.9
22	S-9716/12	795	2.2	1.8
23	S-13582/12	863	2.2	1.9
24	S-12181/12	849	1.9	2.0

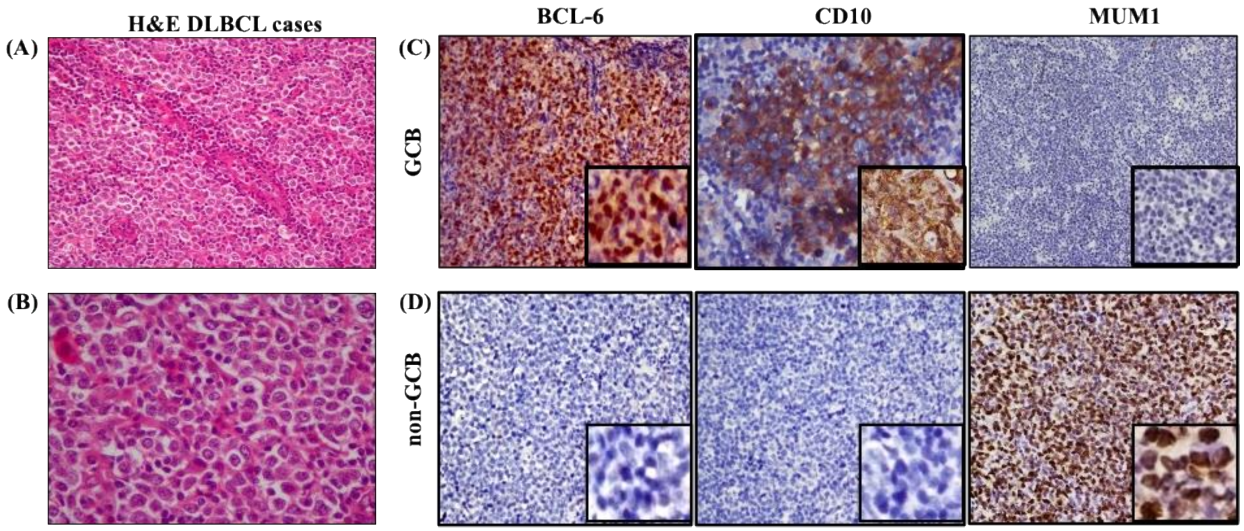

Supplementary Figure S1. The photomicrographs show the immunohistochemical staining profile used for the classification of Diffuse Large B-Cell Lymphoma cases into Germinal Center B-cell like (GCB) and activated B-cell (ABC) subtypes using Han's algorithm. H&E staining in DLBCL cases at **(A)** x200 and **(B)** x400 showing large nuclei. **(C-D)** DLBCL cases were semi-quantitated for BCL-6, CD10, and MUM1 protein expression with a cut-off staining score of ≥30%. **(C)** The GCB phenotype was diagnosed if there was CD10 (membranous) and BCL-6 (nuclear) positivity and a lack of MUM1 expression (top panel). **(D)** Alternatively, the non-GCB subtype lacks CD10 and BCL-6 expression but exhibits MUM1 positivity (bottom panel) (original magnifications: X100 [low] and X400 [high]). Abbreviations: GCB, Germinal Center B-cell like; ABC, Activated B-cell-like; GO, Gene Ontology; DLBCL, Diffuse Large B-Cell Lymphoma; BCL6, B-cell Lymphoma 6; CD10, Cluster of Differentiation 10; MUM1, Multiple Myeloma Oncogene 1.

	A	B	C	D	E	F	G	H	I	J	K	L
1	ADAMTSL1	SPARCL1	STAB1	TMPRSS13	MUC2	IL-4	ADAMTSL1	SPARCL1	STAB1	TMPRSS13	MUC2	IL-4
2	COL5A3	COL5A2	GAS6	CAV1	EGFLAM	IL-3	COL5A3	COL5A2	GAS6	CAV1	EGFLAM	IL-3
3	CPN2	COL13A	LAMC1	MMP9	KDR	IL-2	CPN2	COL13A	LAMC1	MMP9	KDR	IL-2
4	CSN3	HABP2	USH2A	ERG	FN1	IL-10	CSN3	HABP2	USH2A	ERG	FN1	IL-10
5	FBN3	LAMC3	IBSP	THBS1	IL-6	ACTB	FBN3	LAMC3	IBSP	THBS1	IL-6	ACTB
6	COL4A2	TGFB3	LAMB1	SSPO	IL-17	HGDC	COL4A2	TGFB3	LAMB1	SSPO	IL-17	HGD C
7	MUC6	LAMA2	LRRC32	CD14	IFN- γ	RTC	MUC6	LAMA2	LRRC32	CD14	IFN- γ	RTC
8	LRRC4B	LTBP1	MUC5B	PECAM1	TNF	PPC	LRRC4B	LTBP1	MUC5B	PECAM1	TNF	PPC

Supplementary Figure S2: Real-time plate customized to evaluate the expression of forty-four genes (ACTB as housekeeping genes), genomic DNA contamination control, and non-template control (RTC).

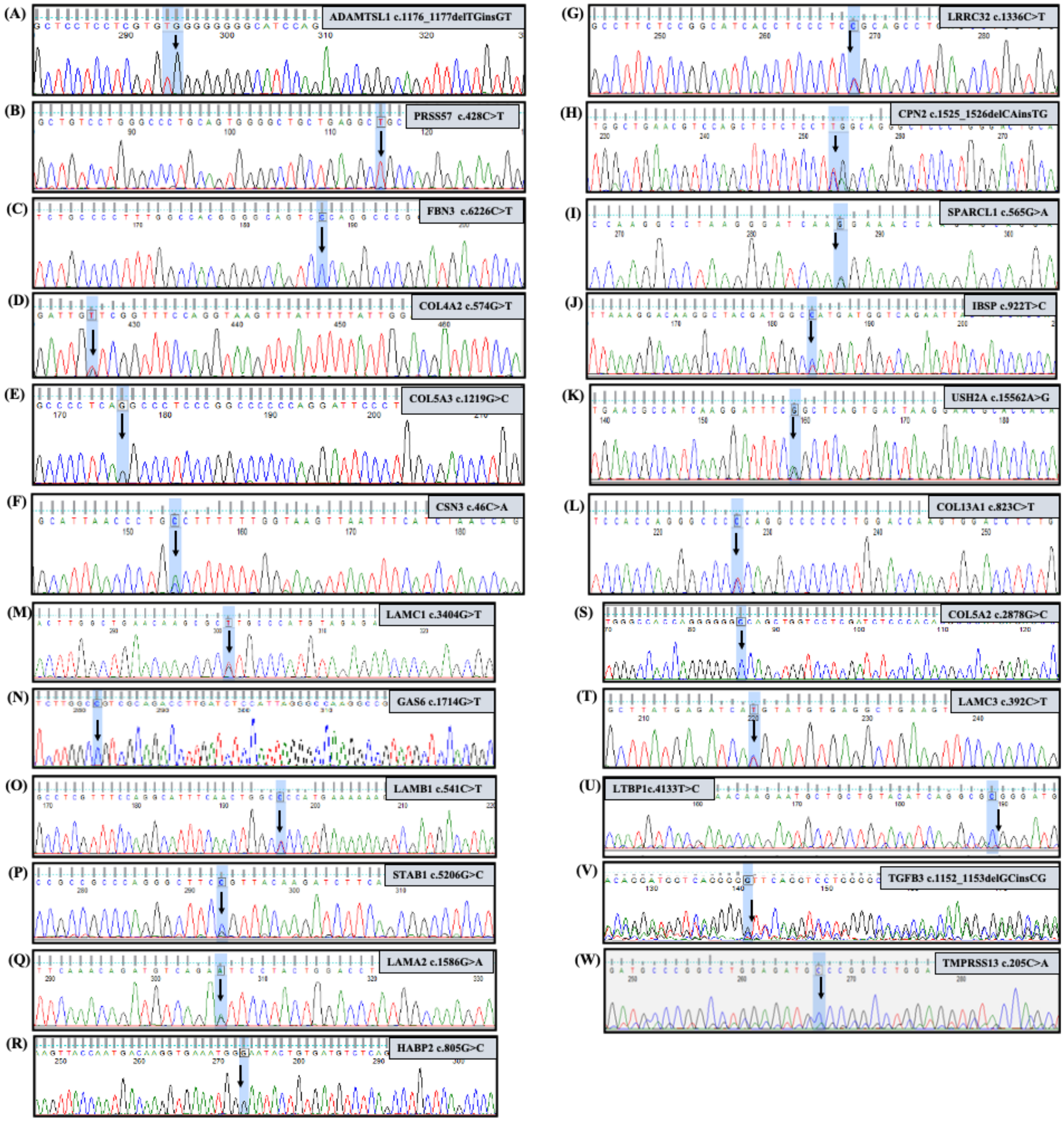

Supplementary Figure S3: (A-W) Sanger validation of stromal mutations identified from whole exome sequencing data.
